# Methyl 3β-methoxy­carbon­yloxy-4,4-di­methyl-17-oxo-16α-(3-oxobut­yl)-16β-carboxylate

**DOI:** 10.1107/S1600536809017243

**Published:** 2009-05-14

**Authors:** Xin Yan, Shiqing Xu, Jingmei Wang, Ying Chen, Peng Xia

**Affiliations:** a Department of Medicinal Chemistry, School of Pharmacy, Fudan University, Shanghai 200032, People’s Republic of China; b Center of Analysis and Measurement, Fudan University, Shanghai 200433, People’s Republic of China

## Abstract

The title steroid, C_29_H_44_O_7_, is a new androgen derivative and a key inter­mediate for synthesizing novel anti-HIV steroid agents. There are four *trans*-fused rings in the structure. The three six-membered rings exhibit chair conformations, while the five-membered ring adopts an envelope conformation.

## Related literature

For discussion of absolute configuration, see: Marker *et al.* (1940 ▶); Fieser & Fieser (1959 ▶); Castro-Méndez *et al.* (2002 ▶). For background to our on-going study synthesizing potential anti-HIV steroid agents, see: Yan *et al.* (2009 ▶).
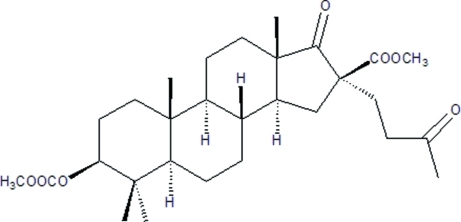


## Experimental

### 

#### Crystal data

C_29_H_44_O_7_
*M* *_r_* = 504.64
Orthorhombic, 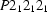

*a* = 8.464 (3) Å
*b* = 9.901 (3) Å
*c* = 32.917 (10) Å
*V* = 2758.4 (14) Å^3^
*Z* = 4
Mo *K*α radiation
μ = 0.09 mm^−1^
*T* = 295 K
0.15 × 0.06 × 0.05 mm

#### Data collection

Bruker SMART APEX CCD area-detector diffractometer
Absorption correction: multi-scan (Blessing, 1995 ▶) *T*_min_ = 0.987, *T*_max_ = 0.996
11219 measured reflections
2846 independent reflections
1785 reflections with *I* > 2σ(*I*)
*R*_int_ = 0.070

#### Refinement

*R*[*F* ^2^ > 2σ(*F* ^2^)] = 0.042
*wR*(*F* ^2^) = 0.089
*S* = 0.86
2846 reflections
332 parameters
H-atom parameters constrained
Δρ_max_ = 0.15 e Å^−3^
Δρ_min_ = −0.16 e Å^−3^

### 

Data collection: *SMART* (Bruker, 2000 ▶); cell refinement: *SAINT* (Bruker, 2000 ▶); data reduction: *SAINT*; program(s) used to solve structure: *SHELXS97* (Sheldrick, 2008 ▶); program(s) used to refine structure: *SHELXL97* (Sheldrick, 2008 ▶); molecular graphics: *SHELXTL* (Sheldrick, 2008 ▶); software used to prepare material for publication: *SHELXL97*.

## Supplementary Material

Crystal structure: contains datablocks I, New_Global_Publ_Block. DOI: 10.1107/S1600536809017243/tk2445sup1.cif

Structure factors: contains datablocks I. DOI: 10.1107/S1600536809017243/tk2445Isup2.hkl

Additional supplementary materials:  crystallographic information; 3D view; checkCIF report

## Figures and Tables

**Fig. 1.**
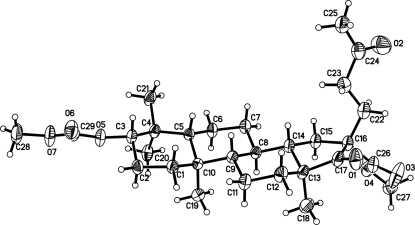
The molecular structure of (I), showing atom labels and 30% probability displacement ellipsoids for non-H atoms.

## supplementary crystallographic information

### Comment

As part of an on-going study synthesizing potential anti-HIV steroid agents (Yan *et al.*,  2009), the title compound (I) was prepared as a key intermediate by Michael addition of 3β-methoxycarbonyloxy-4,4-dimethyl-16β-methoxycarbonyl-androstane-17-one with methyl vinyl ketone in 73.8% yield. Full structural details of (I) are reported herein.

The molecular structure of (I), Fig.1, shows the A/B, B/C and C/D ring junctions to be all *trans*. The cyclohexane rings adopt chair conformations, and the cyclopentane ring adopts an envelope conformation. Based on the known configurations of the methyl groups at the C10 and C13 atoms, see Experimental, the C16-(3'-oxobutyl) group is assigned an α-configuration. The 3-methoxycarbonyloxy and 16-methoxycarbonyl groups are in β-configurations. The stereogenic sites of (I) exhibit the following chirality: C3 = S, C5 = *R*, C8 = *R*, C9 = S, C10 = *R*, C13 = S, C14 = S, and C16 = S.

### Experimental

Methyl vinyl ketone (0.10 ml, 1.24 mmol) was added dropwise to a well stirred mixture of 3β-methoxycarbonyloxy-4,4-dimethyl-16β-methoxycarbonyl-androstane-17-one (214.80 mg, 0.49 mmol) and sodium methoxide (c = 0.5 *M*, 0.50 ml) in methanol (2 ml) under N_2_. The reaction mixture was stirred for 2 h at 293 K and TLC showed the reaction had completed. After adding brine, the reaction mixture was extracted with ethyl acetate and dried over Na_2_SO_4_. The removal of solvent gave a residue which was purified by chromatography on a silica gel column with petroleum ether/ethyl acetate (7:1) as eluent to afford 182.36 mg (73.8%) of (I). Recrystallization from ether to give colorless crystals.

The starting material, 3β-methoxycarbonyloxy-4,4-dimethyl-16β-methoxycarbonyl-androstane-17-one, was derived initially from diosgenin, for which the absolute configurations of all chiral centers of the steroid skeleton have been determined (Fieser & Fieser, 1959; Marker *et al.*, 1940). Recently, the absolute configurations of the chiral centres were confirmed by the X-ray crystal structure determination of a 3-Br substituted steroid substrate synthesized from diosgenin. (Castro-Méndez *et al.*, 2002). Introduction of a side-chain at C16 by Michael addition did not cause inversion of the configurations at C8, C9, C10, C13 and C14. Thus, by comparing the orientation of 16-substitutes to that of methyl groups at C10 and C13, the absolute configuration of (I) could be determined.

### Refinement

All H atoms were placed in the idealized positions with methine C—H = 0.98 Å, methylene C—H = 0.97 Å and methyl C—H = 0.96 Å), and treated as riding with *U*_iso_(H) = 1.2 *U*_eq_(CH_2_ and CH) and 1.5 *U*_eq_(CH_3_). In the absence of significant anomalous scattering effects, 584 Friedel pairs were averaged in the final refinement.

### Crystal data

**Table:**

C_29_H_44_O_7_	*F*(000) = 1096
*M**_r_* = 504.64	*D*_x_ = 1.215 Mg m^−^^3^
Orthorhombic, *P*2_1_2_1_2_1_	Mo *K*α radiation, λ = 0.71073 Å
Hall symbol: P 2ac 2ab	Cell parameters from 700 reflections
*a* = 8.464 (3) Å	θ = 2.4–24.2°
*b* = 9.901 (3) Å	µ = 0.09 mm^−^^1^
*c* = 32.917 (10) Å	*T* = 295 K
*V* = 2758.4 (14) Å^3^	Block, colourless
*Z* = 4	0.15 × 0.06 × 0.05 mm

### Data collection

**Table:**

Bruker SMART APEX CCD area-detector diffractometer	2846 independent reflections
Radiation source: sealed tube	1785 reflections with *I* > 2σ(*I*)
graphite	*R*_int_ = 0.070
phi and ω scans	θ_max_ = 25.2°, θ_min_ = 1.2°
Absorption correction: multi-scan (Blessing, 1995)	*h* = −10→10
*T*_min_ = 0.987, *T*_max_ = 0.996	*k* = −11→10
11219 measured reflections	*l* = −39→31

### Refinement

**Table:**

Refinement on *F*^2^	Primary atom site location: structure-invariant direct methods
Least-squares matrix: full	Secondary atom site location: difference Fourier map
*R*[*F*^2^ > 2σ(*F*^2^)] = 0.042	Hydrogen site location: inferred from neighbouring sites
*wR*(*F*^2^) = 0.089	H-atom parameters constrained
*S* = 0.86	*w* = 1/[σ^2^(*F*_o_^2^) + (0.042*P*)^2^] where *P* = (*F*_o_^2^ + 2*F*_c_^2^)/3
2846 reflections	(Δ/σ)_max_ = 0.001
332 parameters	Δρ_max_ = 0.15 e Å^−^^3^
0 restraints	Δρ_min_ = −0.16 e Å^−^^3^

### Special details

**Table:**

Geometry. All e.s.d.'s (except the e.s.d. in the dihedral angle between two l.s. planes) are estimated using the full covariance matrix. The cell e.s.d.'s are taken into account individually in the estimation of e.s.d.'s in distances, angles and torsion angles; correlations between e.s.d.'s in cell parameters are only used when they are defined by crystal symmetry. An approximate (isotropic) treatment of cell e.s.d.'s is used for estimating e.s.d.'s involving l.s. planes.
Refinement. Refinement of *F*^2^ against ALL reflections. The weighted *R*-factor *wR* and goodness of fit *S* are based on *F*^2^, conventional *R*-factors *R* are based on *F*, with *F* set to zero for negative *F*^2^. The threshold expression of *F*^2^ > σ(*F*^2^) is used only for calculating *R*-factors(gt) *etc*. and is not relevant to the choice of reflections for refinement. *R*-factors based on *F*^2^ are statistically about twice as large as those based on *F*, and *R*- factors based on ALL data will be even larger.

### Fractional atomic coordinates and isotropic or equivalent isotropic displacement parameters (Å^2^)

**Table:**

	*x*	*y*	*z*	*U*_iso_*/*U*_eq_	
O1	0.5861 (3)	0.3685 (3)	1.23879 (6)	0.0805 (9)	
O2	0.1349 (4)	0.0393 (3)	1.19374 (10)	0.1119 (11)	
O3	0.2929 (4)	0.5217 (4)	1.26075 (8)	0.1202 (13)	
O4	0.1781 (3)	0.6056 (3)	1.20619 (7)	0.0641 (7)	
O5	1.0487 (2)	0.4975 (3)	0.93458 (5)	0.0621 (7)	
O6	1.2733 (3)	0.3782 (3)	0.94059 (8)	0.0770 (8)	
O7	1.2090 (3)	0.4978 (3)	0.88526 (6)	0.0845 (9)	
C1	1.0195 (3)	0.4992 (3)	1.04937 (8)	0.0476 (8)	
H1A	1.0288	0.4028	1.0539	0.057*	
H1B	1.0827	0.5448	1.0698	0.057*	
C2	1.0844 (4)	0.5335 (4)	1.00704 (8)	0.0526 (9)	
H2A	1.0776	0.6301	1.0025	0.063*	
H2B	1.1946	0.5073	1.0053	0.063*	
C3	0.9909 (3)	0.4604 (3)	0.97519 (8)	0.0473 (8)	
H3	1.0042	0.3629	0.9790	0.057*	
C4	0.8136 (3)	0.4937 (3)	0.97479 (8)	0.0463 (8)	
C5	0.7499 (3)	0.4752 (3)	1.01902 (7)	0.0394 (7)	
H5	0.7562	0.3779	1.0241	0.047*	
C6	0.5740 (3)	0.5094 (4)	1.02278 (8)	0.0503 (9)	
H6A	0.5599	0.6062	1.0203	0.060*	
H6B	0.5164	0.4664	1.0008	0.060*	
C7	0.5068 (3)	0.4624 (3)	1.06332 (7)	0.0507 (9)	
H7A	0.5052	0.3644	1.0638	0.061*	
H7B	0.3987	0.4939	1.0658	0.061*	
C8	0.6018 (3)	0.5136 (3)	1.09946 (7)	0.0405 (7)	
H8	0.5902	0.6119	1.1012	0.049*	
C9	0.7770 (3)	0.4795 (3)	1.09437 (8)	0.0415 (7)	
H9	0.7816	0.3813	1.0908	0.050*	
C10	0.8464 (3)	0.5411 (3)	1.05431 (8)	0.0393 (7)	
C11	0.8755 (3)	0.5099 (4)	1.13285 (8)	0.0563 (9)	
H11A	0.9816	0.4748	1.1291	0.068*	
H11B	0.8834	0.6070	1.1363	0.068*	
C12	0.8048 (4)	0.4484 (4)	1.17139 (8)	0.0560 (9)	
H12A	0.8089	0.3507	1.1697	0.067*	
H12B	0.8660	0.4765	1.1948	0.067*	
C13	0.6353 (4)	0.4937 (3)	1.17633 (8)	0.0480 (8)	
C14	0.5419 (3)	0.4510 (3)	1.13849 (7)	0.0440 (8)	
H14	0.5571	0.3534	1.1357	0.053*	
C15	0.3684 (4)	0.4715 (4)	1.15094 (8)	0.0511 (9)	
H15A	0.2988	0.4163	1.1344	0.061*	
H15B	0.3377	0.5654	1.1481	0.061*	
C16	0.3623 (4)	0.4270 (4)	1.19609 (9)	0.0511 (9)	
C17	0.5380 (4)	0.4230 (4)	1.20881 (9)	0.0534 (9)	
C18	0.6250 (5)	0.6466 (3)	1.18571 (10)	0.0689 (11)	
H18A	0.6699	0.6968	1.1636	0.103*	
H18B	0.6824	0.6658	1.2102	0.103*	
H18C	0.5164	0.6719	1.1892	0.103*	
C19	0.8356 (4)	0.6959 (3)	1.05608 (10)	0.0581 (9)	
H19A	0.8889	0.7340	1.0330	0.087*	
H19B	0.8846	0.7277	1.0806	0.087*	
H19C	0.7267	0.7228	1.0557	0.087*	
C20	0.7842 (4)	0.6354 (3)	0.95734 (9)	0.0631 (10)	
H20A	0.6739	0.6568	0.9593	0.095*	
H20B	0.8160	0.6375	0.9294	0.095*	
H20C	0.8444	0.7005	0.9724	0.095*	
C21	0.7327 (4)	0.3888 (4)	0.94739 (9)	0.0664 (11)	
H21A	0.7846	0.3866	0.9215	0.100*	
H21B	0.6238	0.4130	0.9437	0.100*	
H21C	0.7392	0.3014	0.9599	0.100*	
C22	0.2843 (4)	0.2896 (4)	1.20214 (10)	0.0631 (10)	
H22A	0.2835	0.2693	1.2310	0.076*	
H22B	0.1752	0.2959	1.1933	0.076*	
C23	0.3614 (4)	0.1732 (4)	1.18004 (10)	0.0636 (10)	
H23A	0.3779	0.1991	1.1519	0.076*	
H23B	0.4643	0.1568	1.1920	0.076*	
C24	0.2683 (5)	0.0443 (4)	1.18101 (10)	0.0699 (11)	
C25	0.3495 (5)	−0.0768 (4)	1.16541 (10)	0.0794 (12)	
H25A	0.2729	−0.1452	1.1592	0.119*	
H25B	0.4214	−0.1101	1.1856	0.119*	
H25C	0.4072	−0.0539	1.1413	0.119*	
C26	0.2757 (4)	0.5230 (4)	1.22479 (11)	0.0627 (10)	
C27	0.0879 (4)	0.6956 (4)	1.23203 (11)	0.0793 (12)	
H27A	0.1585	0.7561	1.2458	0.119*	
H27B	0.0303	0.6436	1.2517	0.119*	
H27C	0.0153	0.7467	1.2157	0.119*	
C28	1.3557 (5)	0.4633 (5)	0.86532 (11)	0.0947 (15)	
H28A	1.3801	0.3701	0.8704	0.142*	
H28B	1.4391	0.5192	0.8757	0.142*	
H28C	1.3453	0.4775	0.8366	0.142*	
C29	1.1861 (4)	0.4489 (4)	0.92210 (10)	0.0558 (9)	

### Atomic displacement parameters (Å^2^)

**Table:**

	*U*^11^	*U*^22^	*U*^33^	*U*^12^	*U*^13^	*U*^23^
O1	0.0667 (18)	0.131 (2)	0.0434 (14)	0.0043 (17)	−0.0064 (13)	0.0247 (15)
O2	0.0654 (19)	0.110 (3)	0.161 (3)	−0.0093 (19)	0.022 (2)	−0.013 (2)
O3	0.146 (3)	0.171 (3)	0.0430 (16)	0.067 (3)	0.0101 (16)	−0.0036 (18)
O4	0.0503 (16)	0.0825 (18)	0.0596 (15)	0.0093 (14)	0.0075 (13)	−0.0078 (14)
O5	0.0458 (13)	0.0985 (19)	0.0421 (12)	0.0121 (14)	0.0106 (10)	0.0020 (12)
O6	0.0684 (18)	0.0835 (19)	0.0793 (18)	0.0202 (16)	0.0232 (15)	0.0053 (15)
O7	0.0592 (16)	0.147 (3)	0.0471 (14)	0.0110 (19)	0.0188 (12)	0.0035 (16)
C1	0.0357 (18)	0.067 (2)	0.0405 (16)	−0.0029 (18)	−0.0008 (13)	−0.0029 (16)
C2	0.0371 (19)	0.070 (2)	0.0506 (18)	−0.0008 (18)	0.0066 (15)	−0.0023 (18)
C3	0.0424 (19)	0.063 (2)	0.0362 (16)	0.0042 (17)	0.0069 (14)	−0.0008 (15)
C4	0.0408 (18)	0.063 (2)	0.0346 (15)	0.0048 (18)	−0.0005 (13)	−0.0026 (16)
C5	0.0377 (17)	0.0461 (19)	0.0346 (15)	0.0012 (16)	−0.0013 (13)	−0.0008 (13)
C6	0.0361 (17)	0.080 (3)	0.0352 (16)	0.0033 (18)	−0.0014 (13)	0.0039 (17)
C7	0.0367 (18)	0.077 (2)	0.0379 (16)	−0.0011 (18)	−0.0036 (14)	−0.0009 (16)
C8	0.0356 (18)	0.049 (2)	0.0366 (15)	0.0028 (16)	0.0005 (13)	−0.0008 (15)
C9	0.0342 (17)	0.052 (2)	0.0379 (16)	0.0012 (16)	−0.0013 (13)	−0.0038 (14)
C10	0.0391 (18)	0.0412 (19)	0.0377 (16)	−0.0014 (15)	0.0035 (13)	−0.0041 (14)
C11	0.0375 (18)	0.092 (3)	0.0398 (17)	0.002 (2)	−0.0044 (14)	−0.0055 (18)
C12	0.047 (2)	0.083 (3)	0.0386 (17)	0.0035 (19)	−0.0093 (15)	−0.0019 (17)
C13	0.0427 (18)	0.068 (2)	0.0333 (15)	0.0025 (18)	−0.0013 (13)	−0.0083 (17)
C14	0.0363 (18)	0.061 (2)	0.0344 (16)	0.0008 (16)	0.0011 (13)	0.0012 (15)
C15	0.0452 (19)	0.071 (2)	0.0373 (16)	0.0047 (19)	0.0028 (14)	0.0039 (16)
C16	0.047 (2)	0.070 (2)	0.0366 (17)	0.0039 (19)	0.0033 (15)	0.0040 (16)
C17	0.051 (2)	0.074 (3)	0.0350 (18)	0.002 (2)	−0.0005 (16)	−0.0020 (17)
C18	0.071 (3)	0.076 (3)	0.059 (2)	0.000 (2)	0.0023 (19)	−0.0143 (19)
C19	0.063 (2)	0.053 (2)	0.058 (2)	−0.0038 (19)	0.0065 (18)	−0.0067 (17)
C20	0.062 (2)	0.083 (3)	0.045 (2)	0.017 (2)	0.0094 (17)	0.0130 (18)
C21	0.055 (2)	0.102 (3)	0.0421 (19)	−0.006 (2)	0.0003 (17)	−0.021 (2)
C22	0.056 (2)	0.085 (3)	0.049 (2)	0.006 (2)	0.0034 (18)	0.0055 (19)
C23	0.059 (2)	0.073 (3)	0.058 (2)	−0.004 (2)	0.0035 (19)	0.0063 (19)
C24	0.070 (3)	0.084 (3)	0.056 (2)	0.004 (3)	0.001 (2)	0.012 (2)
C25	0.101 (3)	0.077 (3)	0.060 (2)	0.001 (3)	0.011 (2)	0.003 (2)
C26	0.061 (3)	0.082 (3)	0.046 (2)	0.007 (2)	0.0127 (18)	0.002 (2)
C27	0.058 (3)	0.092 (3)	0.088 (3)	0.003 (2)	0.016 (2)	−0.025 (2)
C28	0.071 (3)	0.142 (4)	0.071 (3)	0.002 (3)	0.032 (2)	−0.015 (3)
C29	0.045 (2)	0.075 (3)	0.047 (2)	−0.002 (2)	0.0076 (18)	−0.0082 (19)

### Geometric parameters (Å, °)

**Table:**

O1—C17	1.196 (3)	C12—H12A	0.9700
O2—C24	1.205 (4)	C12—H12B	0.9700
O3—C26	1.193 (4)	C13—C17	1.520 (4)
O4—C26	1.314 (4)	C13—C14	1.535 (4)
O4—C27	1.449 (4)	C13—C18	1.547 (4)
O5—C29	1.324 (4)	C14—C15	1.538 (4)
O5—C3	1.470 (3)	C14—H14	0.9800
O6—C29	1.185 (4)	C15—C16	1.551 (4)
O7—C29	1.320 (4)	C15—H15A	0.9700
O7—C28	1.446 (4)	C15—H15B	0.9700
C1—C10	1.531 (4)	C16—C22	1.525 (5)
C1—C2	1.536 (4)	C16—C26	1.528 (5)
C1—H1A	0.9700	C16—C17	1.545 (4)
C1—H1B	0.9700	C18—H18A	0.9599
C2—C3	1.500 (4)	C18—H18B	0.9599
C2—H2A	0.9700	C18—H18C	0.9599
C2—H2B	0.9700	C19—H19A	0.9599
C3—C4	1.536 (4)	C19—H19B	0.9599
C3—H3	0.9800	C19—H19C	0.9599
C4—C20	1.536 (4)	C20—H20A	0.9599
C4—C21	1.537 (4)	C20—H20B	0.9599
C4—C5	1.563 (4)	C20—H20C	0.9599
C5—C6	1.532 (4)	C21—H21A	0.9599
C5—C10	1.563 (4)	C21—H21B	0.9599
C5—H5	0.9800	C21—H21C	0.9599
C6—C7	1.523 (4)	C22—C23	1.511 (5)
C6—H6A	0.9700	C22—H22A	0.9700
C6—H6B	0.9700	C22—H22B	0.9700
C7—C8	1.523 (4)	C23—C24	1.501 (5)
C7—H7A	0.9700	C23—H23A	0.9700
C7—H7B	0.9700	C23—H23B	0.9700
C8—C14	1.514 (4)	C24—C25	1.474 (5)
C8—C9	1.530 (4)	C25—H25A	0.9599
C8—H8	0.9800	C25—H25B	0.9599
C9—C11	1.546 (4)	C25—H25C	0.9599
C9—C10	1.567 (4)	C27—H27A	0.9599
C9—H9	0.9800	C27—H27B	0.9599
C10—C19	1.536 (4)	C27—H27C	0.9599
C11—C12	1.529 (4)	C28—H28A	0.9599
C11—H11A	0.9700	C28—H28B	0.9599
C11—H11B	0.9700	C28—H28C	0.9599
C12—C13	1.511 (4)		
			
C26—O4—C27	116.1 (3)	C13—C14—C15	103.9 (2)
C29—O5—C3	119.0 (3)	C8—C14—H14	106.3
C29—O7—C28	117.1 (3)	C13—C14—H14	106.3
C10—C1—C2	112.2 (2)	C15—C14—H14	106.3
C10—C1—H1A	109.2	C14—C15—C16	104.5 (2)
C2—C1—H1A	109.2	C14—C15—H15A	110.9
C10—C1—H1B	109.2	C16—C15—H15A	110.9
C2—C1—H1B	109.2	C14—C15—H15B	110.9
H1A—C1—H1B	107.9	C16—C15—H15B	110.9
C3—C2—C1	109.8 (2)	H15A—C15—H15B	108.9
C3—C2—H2A	109.7	C22—C16—C26	105.5 (3)
C1—C2—H2A	109.7	C22—C16—C17	111.0 (3)
C3—C2—H2B	109.7	C26—C16—C17	108.1 (3)
C1—C2—H2B	109.7	C22—C16—C15	113.1 (3)
H2A—C2—H2B	108.2	C26—C16—C15	115.6 (3)
O5—C3—C2	109.8 (2)	C17—C16—C15	103.6 (2)
O5—C3—C4	105.3 (2)	O1—C17—C13	127.1 (3)
C2—C3—C4	114.7 (3)	O1—C17—C16	124.2 (3)
O5—C3—H3	109.0	C13—C17—C16	108.6 (3)
C2—C3—H3	109.0	C13—C18—H18A	109.5
C4—C3—H3	109.0	C13—C18—H18B	109.5
C3—C4—C20	111.0 (3)	H18A—C18—H18B	109.5
C3—C4—C21	107.1 (3)	C13—C18—H18C	109.5
C20—C4—C21	109.0 (3)	H18A—C18—H18C	109.5
C3—C4—C5	107.7 (2)	H18B—C18—H18C	109.5
C20—C4—C5	113.6 (2)	C10—C19—H19A	109.5
C21—C4—C5	108.3 (3)	C10—C19—H19B	109.5
C6—C5—C10	110.8 (2)	H19A—C19—H19B	109.5
C6—C5—C4	112.6 (2)	C10—C19—H19C	109.5
C10—C5—C4	117.6 (2)	H19A—C19—H19C	109.5
C6—C5—H5	104.8	H19B—C19—H19C	109.5
C10—C5—H5	104.8	C4—C20—H20A	109.5
C4—C5—H5	104.8	C4—C20—H20B	109.5
C7—C6—C5	111.5 (2)	H20A—C20—H20B	109.5
C7—C6—H6A	109.3	C4—C20—H20C	109.5
C5—C6—H6A	109.3	H20A—C20—H20C	109.5
C7—C6—H6B	109.3	H20B—C20—H20C	109.5
C5—C6—H6B	109.3	C4—C21—H21A	109.5
H6A—C6—H6B	108.0	C4—C21—H21B	109.5
C6—C7—C8	112.7 (2)	H21A—C21—H21B	109.5
C6—C7—H7A	109.1	C4—C21—H21C	109.5
C8—C7—H7A	109.1	H21A—C21—H21C	109.5
C6—C7—H7B	109.1	H21B—C21—H21C	109.5
C8—C7—H7B	109.1	C23—C22—C16	115.5 (3)
H7A—C7—H7B	107.8	C23—C22—H22A	108.4
C14—C8—C7	110.5 (2)	C16—C22—H22A	108.4
C14—C8—C9	109.1 (2)	C23—C22—H22B	108.4
C7—C8—C9	110.7 (2)	C16—C22—H22B	108.4
C14—C8—H8	108.8	H22A—C22—H22B	107.5
C7—C8—H8	108.8	C24—C23—C22	114.3 (3)
C9—C8—H8	108.8	C24—C23—H23A	108.7
C8—C9—C11	112.9 (2)	C22—C23—H23A	108.7
C8—C9—C10	111.7 (2)	C24—C23—H23B	108.7
C11—C9—C10	114.3 (2)	C22—C23—H23B	108.7
C8—C9—H9	105.7	H23A—C23—H23B	107.6
C11—C9—H9	105.7	O2—C24—C25	121.7 (4)
C10—C9—H9	105.7	O2—C24—C23	122.3 (4)
C1—C10—C19	109.3 (3)	C25—C24—C23	116.1 (3)
C1—C10—C5	107.9 (2)	C24—C25—H25A	109.5
C19—C10—C5	114.4 (3)	C24—C25—H25B	109.5
C1—C10—C9	110.0 (2)	H25A—C25—H25B	109.5
C19—C10—C9	109.5 (2)	C24—C25—H25C	109.5
C5—C10—C9	105.5 (2)	H25A—C25—H25C	109.5
C12—C11—C9	113.0 (3)	H25B—C25—H25C	109.5
C12—C11—H11A	109.0	O3—C26—O4	123.1 (4)
C9—C11—H11A	109.0	O3—C26—C16	123.3 (4)
C12—C11—H11B	109.0	O4—C26—C16	113.6 (3)
C9—C11—H11B	109.0	O4—C27—H27A	109.5
H11A—C11—H11B	107.8	O4—C27—H27B	109.5
C13—C12—C11	110.0 (3)	H27A—C27—H27B	109.5
C13—C12—H12A	109.7	O4—C27—H27C	109.5
C11—C12—H12A	109.7	H27A—C27—H27C	109.5
C13—C12—H12B	109.7	H27B—C27—H27C	109.5
C11—C12—H12B	109.7	O7—C28—H28A	109.5
H12A—C12—H12B	108.2	O7—C28—H28B	109.5
C12—C13—C17	117.0 (3)	H28A—C28—H28B	109.5
C12—C13—C14	108.7 (2)	O7—C28—H28C	109.5
C17—C13—C14	99.5 (2)	H28A—C28—H28C	109.5
C12—C13—C18	111.4 (3)	H28B—C28—H28C	109.5
C17—C13—C18	106.2 (3)	O6—C29—O7	126.7 (3)
C14—C13—C18	113.7 (3)	O6—C29—O5	127.0 (3)
C8—C14—C13	113.8 (2)	O7—C29—O5	106.3 (3)
C8—C14—C15	119.5 (2)		
			
C10—C1—C2—C3	−60.6 (3)	C11—C12—C13—C18	−68.1 (3)
C29—O5—C3—C2	73.4 (4)	C7—C8—C14—C13	178.0 (3)
C29—O5—C3—C4	−162.7 (3)	C9—C8—C14—C13	56.1 (3)
C1—C2—C3—O5	177.7 (2)	C7—C8—C14—C15	−58.6 (4)
C1—C2—C3—C4	59.4 (4)	C9—C8—C14—C15	179.5 (3)
O5—C3—C4—C20	−47.5 (3)	C12—C13—C14—C8	−61.2 (4)
C2—C3—C4—C20	73.4 (3)	C17—C13—C14—C8	176.0 (3)
O5—C3—C4—C21	71.4 (3)	C18—C13—C14—C8	63.5 (4)
C2—C3—C4—C21	−167.8 (3)	C12—C13—C14—C15	167.3 (3)
O5—C3—C4—C5	−172.3 (2)	C17—C13—C14—C15	44.5 (3)
C2—C3—C4—C5	−51.5 (4)	C18—C13—C14—C15	−68.1 (3)
C3—C4—C5—C6	178.5 (3)	C8—C14—C15—C16	−166.0 (3)
C20—C4—C5—C6	55.3 (4)	C13—C14—C15—C16	−37.9 (3)
C21—C4—C5—C6	−65.9 (3)	C14—C15—C16—C22	−105.2 (3)
C3—C4—C5—C10	47.8 (4)	C14—C15—C16—C26	133.1 (3)
C20—C4—C5—C10	−75.5 (3)	C14—C15—C16—C17	15.1 (4)
C21—C4—C5—C10	163.3 (3)	C12—C13—C17—O1	25.5 (5)
C10—C5—C6—C7	−58.2 (4)	C14—C13—C17—O1	142.2 (4)
C4—C5—C6—C7	167.8 (3)	C18—C13—C17—O1	−99.5 (4)
C5—C6—C7—C8	52.9 (4)	C12—C13—C17—C16	−152.2 (3)
C6—C7—C8—C14	−173.4 (3)	C14—C13—C17—C16	−35.5 (3)
C6—C7—C8—C9	−52.4 (3)	C18—C13—C17—C16	82.8 (3)
C14—C8—C9—C11	−49.7 (3)	C22—C16—C17—O1	−43.0 (5)
C7—C8—C9—C11	−171.5 (3)	C26—C16—C17—O1	72.2 (5)
C14—C8—C9—C10	179.8 (2)	C15—C16—C17—O1	−164.8 (3)
C7—C8—C9—C10	58.0 (3)	C22—C16—C17—C13	134.7 (3)
C2—C1—C10—C19	−70.6 (3)	C26—C16—C17—C13	−110.1 (3)
C2—C1—C10—C5	54.4 (3)	C15—C16—C17—C13	13.0 (4)
C2—C1—C10—C9	169.1 (2)	C26—C16—C22—C23	−173.8 (3)
C6—C5—C10—C1	178.3 (2)	C17—C16—C22—C23	−57.0 (4)
C4—C5—C10—C1	−50.2 (3)	C15—C16—C22—C23	59.0 (4)
C6—C5—C10—C19	−59.8 (3)	C16—C22—C23—C24	−170.7 (3)
C4—C5—C10—C19	71.8 (3)	C22—C23—C24—O2	9.1 (5)
C6—C5—C10—C9	60.7 (3)	C22—C23—C24—C25	−170.9 (3)
C4—C5—C10—C9	−167.8 (3)	C27—O4—C26—O3	−1.1 (5)
C8—C9—C10—C1	−177.4 (2)	C27—O4—C26—C16	177.7 (3)
C11—C9—C10—C1	52.9 (3)	C22—C16—C26—O3	73.2 (5)
C8—C9—C10—C19	62.4 (3)	C17—C16—C26—O3	−45.6 (5)
C11—C9—C10—C19	−67.3 (3)	C15—C16—C26—O3	−161.0 (4)
C8—C9—C10—C5	−61.2 (3)	C22—C16—C26—O4	−105.5 (3)
C11—C9—C10—C5	169.0 (3)	C17—C16—C26—O4	135.7 (3)
C8—C9—C11—C12	50.8 (4)	C15—C16—C26—O4	20.2 (4)
C10—C9—C11—C12	180.0 (3)	C28—O7—C29—O6	−0.7 (6)
C9—C11—C12—C13	−54.7 (4)	C28—O7—C29—O5	177.7 (3)
C11—C12—C13—C17	169.5 (3)	C3—O5—C29—O6	−1.4 (5)
C11—C12—C13—C14	58.0 (4)	C3—O5—C29—O7	−179.9 (3)
